# How it affects me: the effects of arguments in public debates on marriage equality for young people in Taiwan

**DOI:** 10.3389/fpsyg.2024.1462431

**Published:** 2025-01-22

**Authors:** I-Ching Lee

**Affiliations:** Department of Psychology, National Taiwan University, Taipei, Taiwan

**Keywords:** same-sex marriage, sexual orientation, subjective wellbeing, dyadic sample, debates

## Abstract

How do arguments in public debates regarding same-sex marriage affect young people? The literature has suggested three possible effects. These debates may affect young people, regardless of their sexual identities, due to the fact that young people widely share a set of values pertaining to human rights and equality (H1). These debates may affect sexual minority individuals more strongly than heterosexual individuals because the arguments used in such debates are targeted at sexual minority individuals (H2). Alternatively, these debates may affect positive and negative outcomes in different ways depending on the nature of the arguments (H3). Two experimental studies (*N* = 92 and *N* = 411) were conducted in Taiwan with the goal of testing these three hypotheses. The evidence revealed by these studies largely supported the first hypothesis. The effects observed were similar across young people with different sexual identities and various types of outcomes: reading arguments in support of same-sex marriage increased positive emotions (in both studies) and reduced negative emotions (in Study 2) in comparison with a control condition. Further implications regarding young people's responses to social changes are discussed.

## Introduction

In the past several decades, the restriction of marriage rights to heterosexual couples has been challenged in many countries, especially those in Europe and North America. Taiwan is one of the few countries in Asia that has witnessed extensive debates concerning same-sex marriage (e.g., Chin, 2020; Lee and Lin, 2022; Yeh, 2017). Supporters of same-sex marriage submitted a case to the Constitutional Court that argued for the rights of a same-sex couple. The Constitutional Court ruled in favor of the rights of same-sex couples in May 2017. However, opponents of same-sex marriage launched a massive campaign that resulted in three related referenda (concerning whether the rights of same-sex couples should be protected, whether those rights should be protected in the Civil Code, and whether education in gender equality should be provided at elementary and middle schools) at the end of 2018. The supporters of same-sex marriage were defeated by a margin of approximately 2:1. To comply with the Constitutional Court's ruling (i.e., regarding same-sex couples' right to marry) and the results of the referenda (i.e., the result that the definition of marriage in the Civil Code should be restricted to a union between one man and one woman, which implies that the rights of same-sex couples should be protected in other ways than by changing the Civil Code), the government passed a special law to offer same-sex couples the right to marry. Further information regarding the litigation and legislative actions that occurred before the legalization of same-sex marriage in Taiwan was provided by Kuan (2019).

The corresponding debates concerning same-sex marriage have highlighted the tensions between traditional Chinese culture and modern Taiwan. Traditional Chinese culture has been deeply influenced by Confucianism, which discourages homosexuality on the basis of duties pertaining to filial piety, such as entering marriage and having offspring (e.g., Whyke, 2022; Zhang, 2018). As a result, Adamczyk and Cheng (2015) reported that people in Confucian societies exhibit more disapproval of homosexuality than do people in other countries. However, homosexuality is never directly forbidden in Confucianism, and on one interpretation of the core values of Confucianism (i.e., compassion, tolerance, and kindness), Confucianism could be claimed to support same-sex marriage (Bai, 2021). Coupled with factors that have been reported to predict more liberal attitudes toward homosexuality in other contexts, such as higher levels of economic development (e.g., Stulhofer and Rimac, 2009) and democracy and gender equality (Dion and Diez, 2017; Lee and Hicks, 2011), which are enjoyed in Taiwan, Taiwanese attitudes toward same-sex marriage are expected to be more accepting and supportive. Indeed, in an examination of people's changing attitudes toward homosexuality, Cheng et al. (2016) reported a steady increase in people's tolerance of homosexuality in this context from 1995 to 2012; notably, this increase was much greater than the corresponding increases observed in nearby countries (i.e., China, Japan, and South Korea).

In the context of extensive debates concerning same-sex marriage, the proponents of same-sex marriage have used rights discourse to support same-sex marriage (Lee and Lin, 2022), whereas the opponents of this notion have relied on heteronormative discourse (e.g., gendered appellations, Chin, 2020; hate speech, Yeh, 2017; and heteronormativity, Lee and Lin, 2022) to reject same-sex marriage. According to Lee and Lin (2022), the majority of the arguments that have been made in support of same-sex marriage have claimed that love, marriage, and family are basic human rights and that the legalization of same-sex marriage could provide rights and protections for both same-sex partners and their children. Conversely, the majority of the arguments that have been levied against marriage equality focus on heterosexuality as the basis of marriage and family, the negative consequences of the legalization of same-sex marriage, and the fact that the negative consequences of such legalization entail that a social consensus must be reached before legalization can occur (Lee and Lin, 2022).

How do the arguments that have been made as part of these debates concerning same-sex marriage affect the general public, especially young people? Young people are the focus of this research because they are in the process of developing intimate relationships and consolidating their identities (Benson and Elder, 2011). For example, Benson and Elder examined young adults by reference to various indices (e.g., sexual experiences and psychosocial maturity, including autonomy and social responsibility). These authors reported that less than one-third of the young adults in their sample were classified to the “early adults” cluster, which was characterized by higher levels of psychosocial maturity, social maturity, and adult responsibilities. Asian individuals were more likely to be classified in the “late adults” cluster, which was characterized by relatively low levels of psychosocial maturity, social maturity, and adult responsibilities. In addition, Lee et al. (in press) interviewed young people regarding their intimate relationships and reported that heteronormative contexts often impact young people's understanding of themselves. On the basis of the meanings that people attach to their actual experiences, young people gradually come to understand themselves and formulate their sexual identities. The arguments that have been made as part of the debates concerning same-sex marriage may hurt the feelings of young people in Taiwan because the majority of people who are younger than 30 (82.9%) in Taiwan have been reported to support same-sex marriage (as observed among a representative sample in Taiwan, Huang and Chang, 2018). Following the defeat of the pro-same-sex marriage position in the aforementioned referenda, it was reported that some sexual minority individuals (i.e., lesbian, gay, bisexual, queer, transgender, and questioning individuals) took their own lives (e.g., Wong, 2018). Following these referenda, a substantial portion of participants in relevant research reported that public debates concerning same-sex marriage had significant impacts on their occupational or academic performance (39.5%), friendships (34.2%), relationships with their families (37.7%), and mood or sleep quality (57.4%, Lin et al., 2020). Lin et al. reported that sexual minority individuals were more likely to report being affected by these public debates than were heterosexual individuals, with odds ratios ranging from 2.06 to 2.87.

Despite the news and these self-evaluations, experimental evidence regarding the causal effects of debates concerning same-sex marriage on the mental health of people in Taiwan and elsewhere remains lacking. Researchers have used cross-sectional data (e.g., data concerning depression and anxiety among a representative sample in the Netherlands, Chen and Van Ours, 2022; data regarding insomnia, anxiety, hostility, depression, and mental health status among two convenience samples in Taiwan, Chen et al., 2021; or data pertaining to the subjective wellbeing of people in same-sex relationships among a representative sample in England and Wales, Boertien and Vignoli (2019) and longitudinal survey data concerning mental health [e.g., physical and mental health data from the United States (U.S.), Hatzenbuehler et al., 2010, 2012, or data pertaining to intimate relationships in Vermont, Balsam et al., 2008] to examine the effects of the legalization (or prohibition) of same-sex marriage. On the basis of comparisons of the evidence before and after various legal changes to same-sex marriage, these researchers have reported that the legalization (or prohibition) of same-sex marriage may ameliorate (or exacerbate) the problems faced by sexual minority individuals (for an overview of relevant studies, see Drabble et al., 2021; e.g., on subjective wellbeing, see Boertien and Vignoli, 2019; on depression and anxiety, see Chen and Van Ours, 2022; on mental and physical problems, see Chen et al., 2021; on physical and mental health, see Hatzenbuehler et al., 2010, 2012; and on relationship dissolution, see Balsam et al., 2008).

Although these survey data have provided some causal evidence regarding the effects of policy changes, these previous studies have exhibited four limitations. First, the different levels of support for same-sex marriage or different rates of mental health indicators observed among the convenience samples investigated in this research before and after the relevant policy changes may have been the result of sampling errors, attrition, or actual changes. The violation of the assumption of independence prevents direct comparisons of the two time points on the basis of cross-sectional data (e.g., Lin et al., 2019). Second, cross-sectional data obtained from a representative sample before and after policy changes reveal potential effects at the population level rather than at the individual level. It is possible that such policy changes affect only a portion of the population. Third, because policy changes represent only some of the many events that occur, researchers who compare longitudinal data before and after policy changes find it difficult to determine precisely whether the effects observed in this context are solely the result of policy changes. In addition, the effects of same-sex marriage may be to the result of debates concerning same-sex marriage before legalization, the symbolic meanings of the legalization passed concerning same-sex marriage, or the legal rights offered to same-sex couples following the legalization of same-sex marriage. It is important to distinguish among the sources of these effects. Finally, evidence regarding the effects of same-sex marriage has been reported mainly in the U.S. and among white populations (see Drabble et al., 2021). The present research aims to examine experimental evidence pertaining to the effects of arguments in public debates concerning same-sex marriage on young people in Taiwan.

Previous research and theories have proposed three hypotheses regarding the effects of public debates concerning same-sex marriage: (1) such debates have a general effect regardless of perceivers' sexual orientation; (2) such debates have a specific effect on sexual minority individuals; and (3) such debates have differential effects in terms of positive and negative outcomes. Because the arguments made both for and against same-sex marriage in Taiwan are largely similar to those that have been made elsewhere (Lee and Lin, 2022), the hypotheses proposed in this research were developed on the basis of the assumption of cultural similarity between evidence collected in Taiwan and evidence obtained elsewhere.

First, arguments in public debates may affect young people regardless of their sexual orientation because same-sex marriage is widely viewed as an issue pertaining to human rights (Lee and Lin, 2022), and most young people support same-sex marriage (Lee, 2020; Lin, 2020). Berggren et al. (2018) examined cross-national data and reported that legal recognition of partnership, marriage, and adoption rights for same-sex couples is positively related to general life satisfaction. In addition, indirect evidence has been reported in previous research, indicating that when people support human rights in principle, they are unlikely to support violations of the human rights of outgroup members (including with regard to the views of heterosexual individuals on same-sex marriage in Taiwan, Yen et al., 2020). Thus, Hypothesis I posits that arguments for same-sex marriage may increase the subjective wellbeing of young people, regardless of their sexual orientation, whereas arguments against same-sex marriage may reduce their subjective wellbeing.

Second, according to minority stress theory (which posits that people may experience stress as a result of their sexual minority status; Meyer, 2003; Frost and Meyer, 2023) and the literature on intergroup relationships (which has focused on, e.g., health disparities among sexual minority individuals; Ryan et al., 2017), sexual minority individuals may be affected by particular arguments. The arguments that have been levied against same-sex marriage may signal a societal environment that is hostile to individuals who are not viewed as normative (e.g., sexual minority individuals) while protecting those who are viewed as normative (e.g., heterosexual individuals, Frost and Meyer, 2023). The distinction between the ingroup (heterosexual) and the outgroup (sexual minority) in an environment in which heterosexism is viewed as proper and normative may prevent heterosexual individuals from being hurt by such arguments. For example, heterosexual men may assert normative heterosexuality while engaging in gender-threatening activities as a way of reducing their anxiety (e.g., Pinel et al., 2019; Prewitt-Freilino and Bosson, 2008); however, this coping strategy is not available to sexual minority individuals. Previous research on the impacts of public debates on same-sex marriage has seemed to suggest a specific effect on sexual minority individuals (e.g., a stronger association between debate-related stress and psychological distress among sexual minority individuals than among heterosexual allies in Australia, Ecker et al., 2019, or the observation of significant exposure to stress among sexual minority individuals but not among heterosexual individuals in the U.S., Flores et al., 2018). It is thus unsurprising that researchers have tended to focus on sexual minority individuals in their efforts to document the impacts of same-sex marriage (e.g., in Australia, Casey et al., 2020; in the U.S., Frost and Fingerhut, 2016; Rostosky et al., 2009). Thus, Hypothesis II posits that arguments in support of same-sex marriage may increase the subjective wellbeing of young sexual minority individuals, whereas arguments against same-sex marriage may reduce their subjective wellbeing.

Finally, it is possible that public debates concerning same-sex marriage have differential effects on positive and negative indicators. Previous researchers have reported that sexual minority individuals are more likely to exhibit psychopathology or negative affect in response to perceived discrimination; however, this pattern has not been observed with respect to positive indicators (e.g., positive affect or life satisfaction; Douglass et al., 2017, as well as mental health and subjective wellbeing; Garrison et al., 2018). However, the evidence that has been reported in this regard has been inconsistent to some degree; namely, homophobia observed in neighborhoods has been reported to reduce psychological wellbeing, which is a positive indicator (Kavanaugh et al., 2020). The various effects of perceived hostility in the environment on different aspects of subjective wellbeing that have been reported in previous research may be the result of an evaluative fit mechanism or a congruence principle (Paolini and McIntyre, 2019). According to such an evaluation fit mechanism or a congruence principle, if public debates are perceived as a form of discrimination, they may exacerbate negative indicators of psychological wellbeing. Conversely, if public debates are perceived as indicating support for human rights, they may promote positive indicators of psychological wellbeing. Thus, Hypothesis III posits that the effects of such arguments differ between positive (e.g., life satisfaction and positive affect) and negative (e.g., negative affect) indicators of subjective wellbeing.

Hypotheses I and II are based on the assumption that perceived hostility (acceptance) in the environment may prevent (promote) young people from accepting themselves and exhibiting subjective wellbeing. The distinction between these two hypotheses lies in whether the arguments are perceived to be directed against humans in general (e.g., by targeting human rights, equality for everyone, or all individuals who support such a belief) or against a specific group (i.e., sexual minority individuals). Hypothesis III is based on the assumption that positive indicators are more sensitive to the effects of perceived acceptance in the environment, whereas negative indicators are more sensitive to the effects of perceived hostility in the environment. Although previous studies on the impacts of debates concerning same-sex marriage have seemed to be more consistent with the second hypothesis, all three potential hypotheses were tested at two time points before the legalization of same-sex marriage in Taiwan.

### Current studies

To determine how the arguments that have been made as part of debates concerning same-sex marriage affect young people's subjective wellbeing, two experimental studies were conducted; these studies involved manipulating the various arguments (i.e., supporting arguments and opposing arguments) that were extracted from public debates concerning same-sex marriage in Taiwan (Lee and Lin, 2022). Both studies followed all the research ethics codes of the university, and ethical approval was issued by National Cheng-chi University [NCCU-REC-201505-I008]. Hypothesis I predicts that the effects of the arguments that have been made in debates concerning same-sex marriage affect young people in general. That is, regardless of their sexual orientation, participants are expected to report higher levels of subjective wellbeing in the yes condition (which featured supportive arguments) than in the control condition and to report lower levels of subjective wellbeing in the no condition (which featured opposing arguments) than in the control condition. Hypothesis II predicts that the effects of the arguments that have been made in debates concerning same-sex marriage affect only young sexual minority individuals. Young sexual minority individuals (but not young heterosexual individuals) are thus expected to report higher levels of subjective wellbeing in the yes condition than in the control condition and to report the lowest levels of subjective wellbeing in the no condition. Hypothesis III predicts differential effects with respect to positive and negative indicators of subjective wellbeing. In comparison with the control condition, arguments in support of same-sex marriage may increase positive indicators of subjective wellbeing (e.g., life satisfaction and positive emotions), whereas arguments against same-sex marriage may increase negative indicators of subjective wellbeing (e.g., negative emotions). Although these studies were not preregistered, the data and materials of both studies are uploaded at https://reurl.cc/G5G9dx to offer public access.

## Study 1

### Procedure

In the literature, people with different sexual orientations have often been recruited from different sources (e.g., sexual minority individuals have been recruited from the sexual minority community; Rostosky et al., 2009) that may differ in terms of demographic characteristics, or participants may be recruited from the same sources, which may result in a heterosexual sample that is much larger than the sexual minority sample. To ensure that the relative sizes of the samples of heterosexual and sexual minority individuals remained similar and to control for the different backgrounds of the participants, I drew insights from the paired sample design employed by Balsam et al. (2008) and conducted this study on the basis of a paired sample. Advertisements were posted on school Facebook pages and sexual minority-related boards hosted on sites associated with the bulletin board system (BBS), which are web spaces that are commonly frequented by young people. Because women may exhibit more favorable attitudes toward homosexuality than do men (e.g., Adamczyk and Cheng, 2015), the participants were asked to pair themselves with a same-sex friend who had a different sexual orientation from their own if possible.

This experiment was conducted online, and the participants first read an information sheet concerning the experiment before providing their consent to participate in this research. They were asked to respond while alone. The pairs of participants were randomly assigned to read the same essay, which featured arguments in support of marriage equality (in the yes condition), arguments against marriage equality (in the no condition), or incidents threatening food safety (in the control condition). Because previous researchers have documented the health disparities exhibited by sexual minority individuals (Ryan et al., 2017), before the participants read the essay, they reported their *trait* subjective wellbeing, thus allowing this research to control for potential disparities. After the participants read the news, they reported their views concerning marriage equality and their *state* subjective wellbeing, thus capturing changes in subjective wellbeing that occurred after the participants read the essay. The data referenced in this research were collected in 2015 (in particular, the first case was collected on November 16, while the final case was collected on March 19 of the following year), when the legalization of same-sex marriage had begun to attract attention in Taiwan.

### Participants

Because previous researchers have suggested that the effects of public debates among sexual minority individuals (e.g., between debate-related stress and psychological distress, *r* = 0.53 in Australia, Ecker and Lykins, 2023) are characterized by large effect sizes, G^*^power software was used to estimate a sample size; this estimation indicated that a sample of *n* = 36 was necessary for this research. It was thus decided to recruit at least 36 pairs of individuals for this study.

Forty-nine pairs of friends were invited to participate in an online experiment. Most of the participants followed the instructions for this study by asking a same-sex friend to participate in the experiment as well (87.8%). Most of the pairs included a heterosexual friend paired with a lesbian or gay friend (i.e., LG friend, 47.8%) or a heterosexual friend paired with a bisexual friend (32.6%). The average age of participants was 22.6 years, and their ages ranged overall from 18 to 32 years. The majority of the participants were female (57.1%), Minnanese (66.7%), not religious (57.7%), and resided in Taipei (38.6%). None of the participants in this research were transgender individuals.

### Manipulation

The essays employed in the experimental conditions were developed on the basis of arguments drawn from Lee and Lin (2022); in these essays, arguments either in support of or against same-sex marriage were content analyzed from news stories. The essay used in the yes condition included arguments rooted in human rights discourse, whereas the essay used in the no condition included arguments pertaining to heterosexual favoritism (see the Appendix). Because public debates and social changes may be perceived negatively in Taiwan, a negative phenomenon that occurred during the same time, i.e., incidents threatening food safety, was selected for the control condition (see the Appendix). All three essays exhibited the same word count in Chinese.

### Measures

#### Attention check

An item was included to ask the participants to indicate the main theme of the article that they had read. Four options were offered: the government should be responsible for issues pertaining to food safety, the government should not be responsible for issues pertaining to food safety, same-sex marriage should be legalized, and same-sex marriage should not be legalized. Six participants did not respond to the attention check item correctly and were thus excluded; this group included one participant in the control condition, two participants in the yes condition, and three participants in the no condition.

#### Subjective wellbeing scale

*Trait* subjective wellbeing was evaluated by reference to participants' cognitive evaluations of life and their positive and negative emotions before they read the essay. The items were scored on a five-point scale ranging from 1 (*strongly disagree*) to 5 (*strongly agree*). Participants' cognitive evaluations of life were measured via four items drawn from the Satisfaction with Life Scale (Diener et al., 1985; translated by Wu and Yao, 2006). In addition, five items pertaining to positive emotions and five items pertaining to negative emotion were drawn from Diener et al. (1995); these items have been translated by previous researchers (Chien et al., 2009). All the scales exhibited good reliability (α = 0.75 for satisfaction with life, α = 0.89 for positive emotions, and α = 0.77 for negative emotions). The same subjective wellbeing scale was used after the participants read the essay; however, at this time, the score was measured at the *state* level. The instructions asked the participants to indicate how they felt *at the current moment*. Three scores were calculated: life satisfaction, positive emotions, and negative emotions. Higher scores indicated better subjective wellbeing. Change scores were also calculated, such that higher scores indicated better subjective wellbeing.

#### Attitudes toward marriage equality

One item was adapted from the 2012 Taiwan Social Change Survey (Chang et al., 2014), one item was drawn from the Taiwan Family and Marriage Survey (Chunghua 21st Century Think Tank, 2013), and one item was developed by the author (i.e., I support the legalization of same-sex marriage). Participants responded to these items on a scale ranging from 1 (strongly disagree) to 6 (strongly agree); the higher the average score was, the greater the degree of support for marriage equality. The reliability of this measure was excellent (a = 0.99).

#### Demographic information

The participants reported their sexual orientation, age, sex, religion, and area of residence.

A measure of participants' views concerning intimate relationships was also included in this study; however, it was not related to the current research and was not analyzed in further detail.

## Results and discussion

To check for nonindependent responses within the dyads, an analysis of the degree of independence pertaining to the dependent variable was conducted (Kenny, 1995). Because the pairs were distinguishable on the basis of their role (i.e., whether they volunteered to participate in the study or were recruited by a friend), their *trait* and *state* subjective wellbeing and attitudes toward same-sex marriage were explored by reference to Pearson *r*s to check for nonindependence. Life satisfaction and positive emotions were not correlated within the pairs [before reading the essay, 0.04 > *r*s (*k* = 45) > −0.17, *p*s > 0.27; after reading the essay, 0.12 > *r*s (*k* = 45) > −0.08, *p*s >0.43], with the exception of negative emotions following manipulation, where *r* (*k* = 45) = 0.31, *p* = 0.04. Attitudes toward same-sex marriage were also related within the pairs [*r* (*k* = 45) = 0.42, *p* = 0.004]. In addition, attitudes toward same-sex marriage did not differ by role (*M*_volunteers_ = 5.72 vs. *M*_recruited_ = 5.69, *p* = 0.83). However, a marginal conditional effect on attitudes toward same-sex marriage was observed (see the fourth row in Table 1). Attitudes toward same-sex marriage were controlled for in subsequent analyses.

**Table 1:** Means and confidence intervals by condition: Studies 1 and 2.

**Measures**	**Conditions**			***F* tests**
	**Yes**	**No**	**Control**	
**Attitudes toward same-sex marriage (1–6)**				
Study 1	6.00 (5.67, 6.33)^a^	5.57 (5.31, 5.82)^b^	5.55 (5.34, 5.76)^b^	2.87^+^
Study 2	5.57 (5.25, 5.90)	5.59 (5.15, 6.03)	5.58 (5.16, 6.00)	0.002
**Life satisfaction (change scores)**				
Study 1	−0.20 (−0.47, 0.07)	−0.17 (−0.36, 0.04)	−0.11 (−0.27, 0.06)	0.21
Study 2	−0.25 (−0.40, −0.11)	−0.33 (−0.51, −0.15)	−0.42 (−0.59, −0.24)	1.04
**Positive emotions (change scores)**				
Study 1	0.23 (−0.15, 0.61)^a^	−0.33 (−0.62, −0.05)^b^	−0.29 (−0.52, −0.05)^b^	3.11^+^
Study 2	−0.07 (−0.38, 0.23)^a^	−1.55 (−1.94, −1.17)^b^	−1.08 (−1.45, −0.72)^b^	19.55^***^
**Negative emotions (change scores)**				
Study 1	0.55 (0.15, 0.95)	0.16 (−0.15, 0.46)	0.34 (0.09, 0.59)	1.26
Study 2	0.55 (0.28, 0.83)^a^	−0.44 (−0.79, −0.09)^b^	−0.40 (−0.74, −0.07)^b^	13.60^***^

*Post hoc* comparisons were conducted if the F tests indicated at least marginal significance. Different letters in the subscripts indicate differences as of the *post hoc* comparison (ps < 0.05).

^+^p < 0.07.

^***^p < 0.001.

A mixed-effects model (which was employed to control for the dyadic effect) was used to test the effects of condition (i.e., the yes, no, or control condition) and sexual orientation (i.e., heterosexual or sexual minority) on the three indicators of subjective wellbeing after controlling for participants' sex and attitudes toward same-sex marriage. Before the participants read the essay, neither the effects of condition (*p*s > 0.30) nor the interaction effects between condition and sexual orientation (*p*s > 0.37) were significant.

The same mixed-effects model was used to investigate the three indicators pertaining to change scores of subjective wellbeing. The evidence supported Hypothesis I because young people, regardless of their sexual orientation, were observed to be affected by arguments in support of same-sex marriage. A marginally significant effect of condition [*F*_(2,43.76)_ = 3.11, *p* = 0.054; see the mean scores and confidence intervals presented in Table 1] on the change scores pertaining to positive emotions was observed. The *post hoc* tests revealed that participants in the yes condition reported more positive emotions than did those in the other conditions (between the yes and no conditions, *d*+ = 0.70, *p* = 0.03; between the yes and control conditions, *d*+ = 0.67, *p* = 0.03). Participants in the no condition and the control condition did not differ in terms of the change scores pertaining to positive emotions (*p* = 0.81). The effect of condition and the interaction effect were not significant with respect to the other indicators (*p*s > 0.29).

Although overwhelming support for same-sex marriage was observed among young people, the level of support exhibited by heterosexual individuals was lower than that exhibited by sexual minority individuals (*d*+ = −0.42, *p* = 0.01; see Table 2). In addition, across conditions, heterosexual individuals reported higher levels of life satisfaction (*d*+ = 0.54, *p* = 0.02) and fewer negative emotions (*d*+ = 0.43, *p* = 0.02) than did sexual minority individuals before the manipulation (see Table 2). After the manipulation, regardless of the condition, heterosexual individuals reported lower life satisfaction than did sexual minority individuals (*d*+ = −0.59, *p* = 0.02), whereas sexual minority individuals tended to report fewer positive emotions than did heterosexual individuals (*d*+ = 0.36, *p* = 0.09; see Table 2). No other effects were significant (*p*s > 0.15).

**Table 2:** Means and confidence intervals by sexual orientation: Studies 1 and 2.

**Measures**	**Sexual orientation**				**F tests**
	**Heterosexual**	**LG**	**Bisexual**	**Other**	
**Attitudes toward same-sex marriage (1–6)**					
Study 1	5.56 (5.37, 5.76)^a^	5.85 (5.67, 6.03)^b^			6.87^*^
Study 2	5.07 (4.93, 5.21)^a^	5.82 (5.33, 6.32)^b^	5.85 (5.41, 6.30)^b^	5.35 (4.98, 5.72)^c^	6.18^***^
**Life satisfaction (before manipulation, 1–6)**					
Study 1	4.08 (3.80, 4.36)^a^	3.62 (3.37, 3.88)^b^			5.73^*^
Study 2	4.12 (4.00, 4.24)	3.96 (3.53, 4.38)	4.14 (3.76, 4.53)	3.77 (3.45, 4.09)	1.43
**Life satisfaction (change scores)**					
Study 1	−0.31 (−0.49, −0.13)^a^	−0.002 (−0.17, 0.16)^b^			6.20^*^
Study 2	−0.31 (−0.38, −0.25)	−0.34 (−0.59, −0.10)	−0.43 (−0.65, −0.21)	−0.25 (−0.43, −0.06)	0.53
**Positive emotions (before manipulation, 1–6)**					
Study 1	4.33 (4.08, 4.58)	4.13 (3.91, 4.36)			1.26
Study 2	4.24 (4.13, 4.36)	4.18 (3.78, 4.59)	4.23 (3.86, 4.60)	4.19 (3.88, 4.50)	0.05
**Positive emotions (change scores)**					
Study 1	0.02 (−0.23, 0.28)	−0.28 (−0.51, −0.05)			3.01+
Study 2	−1.05 (−1.20, −0.90)	−0.76 (−1.28, −0.24)	−0.92 (−1.39, −0.45)	−0.88 (−1.27, −0.48)	0.59
**Reverse coded: negative emotions (before manipulation, 1–6)**					
Study 1	4.19 (3.96, 4.43)^a^	3.81 (3.59, 4.02)^b^			6.09^*^
Study 2	4.35 (4.23, 4.48)	4.16 (3.72, 4.60)	4.05 (3.66, 4.45)	4.19 (3.86, 4.52)	1.01
**Negative emotions (change scores)**					
Study 1	0.29 (0.04, 0.53)	0.41 (0.19, 0.64)			0.70
Study 2	−0.14 (−0.28, −0.01)	−0.32 (−0.79, 0.16)	0.03 (−0.40, 0.46)	0.05 (−0.31, 0.40)	0.68

Owing to the small sample size investigated in Study 1, individuals who identified as lesbian, gay, bisexual, or other were combined. LG: lesbian and gay. *Post hoc* comparisons were conducted if the *F* tests indicated at least marginal significance. Different letters in the subscripts indicate differences as of the *post hoc* comparison (ps < 0.05). ^+^*p* < 0.10; ^*^p < 0.05, ^***^p < 0.001.

Because no pairing effects were observed with respect to participants' life satisfaction or positive emotions in Study 1, Study 2 was conducted to include a larger sample without pairing with the aim of exploring the effects of public debates in further detail. This larger sample facilitated an examination of differences among sexual minority individuals in this context.

## Study 2

The study was conducted in 2017 (from June to October), when some cities and counties in Taiwan already allowed same-sex couples to register their same-sex unions (with very limited legal rights). Participants were recruited individually from the same sources as used in Study 1 and asked to respond to an online experiment in the same manner as in Study 1. The contrast between the effects of the yes condition and the control condition on positive emotions was characterized by an effect size larger than 0.66 according to G^*^power. Therefore, to achieve a power of 0.90 and detect an interaction effect between condition and sexual orientation (numerator df = 6), a sample size of 134 was required.

### Participants

A total of 411 participants (277 women) passed the manipulation and attention checks. Among these participants, 278 (67.6%) identified as heterosexual, while the others identified as bisexual (13.4%), lesbian or gay (8.0%), or other (11.0%, 9.5% of whom identified as questioning). The average age of participants was 19.54 years, and their ages ranged from 18 to 24 years.

### Measures

The sequence of the measures was the same as used in Study 1. The same essays and measures that were employed in Study 1 (subjective wellbeing scale, αs > 0.74; attitudes toward marriage equality, α = 0.98) were measured once again in Study 2, albeit with some adjustments. The items used to measure the dependent variable, i.e., subjective wellbeing, were reduced (namely, to four items for life satisfaction and three items each for positive and negative emotions).

### Results and discussion

To determine the equivalence of participants in the assigned conditions, a multivariate analysis of variance (MANOVA) was conducted. The effects of gender, condition, sexual orientation (i.e., heterosexual, lesbian or gay, bisexual, or other), and their interaction effects on the three indicators of subjective wellbeing *before* the manipulation were examined. Before the manipulation, no omnibus effect of condition (*p* > 0.84) or any other omnibus effects (*p*s > 0.13) were observed.

The same MANOVA was conducted to investigate the three indicators of subjective wellbeing (i.e., change scores pertaining to life satisfaction, positive emotions, and negative emotions) with the goal of exploring the effects of gender, condition, and sexual orientation following the manipulation. As in Study 1, condition was observed to impact the affective indicators of subjective wellbeing (see Table 1). The participants reported significantly better subjective wellbeing (i.e., higher levels of positive emotions and lower levels of negative emotions) in the yes condition than in the control condition (positive emotions, *d*+ = 0.50, *p* < 0.001; negative emotions, *d*+ = 0.52, *p* < 0.001) or the no condition (positive emotions, *d*+ = 0.74, *p* < 0.001, negative emotions, *d*+ = 0.54, *p* < 0.001), with the exception of a marginal omnibus effect of gender (*p* = 0.07) that indicated that women (*M* = −0.44) exhibited lower levels of life satisfaction than did men across conditions (*M* = −0.23, *d*+ = −0.22, *p* < 0.001). No other effects approached the level of significance (*p*s > 0.35).

### Supplementary analysis

To test the effects of gender, sexual orientation, and condition on attitudes toward same-sex marriage (collected after the manipulation) in further detail, an ANOVA was conducted. Only a significant main effect of sexual orientation was detected, as was observed in Study 1 (Table 2). Despite the fact that heterosexual participations reported general support for same-sex marriage, they supported same-sex marriage to a lesser degree than did LG participants (*d*+ = −0.51, *p* = 0.004) or bisexual participants (*d*+ = −0.53, *p* = 0.001); however, the difference between participants with other identities and bisexual participants was marginal (*d*+ = −0.34, *p* = 0.089). No other *post hoc* contrasts (*p*s > 0.13) or other effects (*p*s > 0.25) were significant.

## General discussion

The current research revealed how experiments can provide insights into social phenomena by examining the effects of public debates concerning same-sex marriage on young people through the presentation of arguments drawn from newspapers in Taiwan (Lee and Lin, 2022). Despite the fact that the news that emerged following the referenda on same-sex marriage in Taiwan suggested that sexual minority individuals suffered a great deal, the present research revealed that young heterosexual people were also affected by these debates, thus supporting Hypothesis 1 but not Hypothesis 2. Because the effect of condition was most robust for positive emotions (Studies 1 and 2) and somewhat robust for negative emotions (Study 2), Hypothesis 3, which proposed an evaluative fit mechanism in this context, was not supported.

As these debates progressed, their effects on young people may have been exacerbated. In Study 1, which was conducted in 2015, the arguments were revealed to affect participants' positive *state* emotions. In Study 2, which was conducted in 2017, when some form of same-sex union had already been recognized by local governments, the arguments affected participants' positive and negative *state* emotions (see the findings reported in the discussions of these two studies). These findings suggest that people who share such values and beliefs should be included in investigations of various situations that may challenge widely accepted human rights, values, or equality. For example, Mallett et al. (2008) reported that heterosexual individuals who read a summary of events pertaining to a hate crime committed against sexual minority individuals on campus might spontaneously take the perspective of sexual minority individuals and feel anger toward the school administration. The more these heterosexual individuals exhibited such spontaneous sexual minority perspective-taking, the more they reported participating in activities aimed at improving the status of sexual minority individuals (Mallett et al., 2008).

These findings are different from those that have been reported in Australia (Ecker et al., 2019) and the U.S. (Flores et al., 2018), in which context same-sex debates have been demonstrated to have differential effects on heterosexual individuals and sexual minority individuals. One possible explanation for this differences is that previous researchers have investigated representative samples rather than young people. Members of younger generations have been reported to accept homosexuality at higher rates than members of older generations (e.g., Adamczyk and Cheng, 2015). It is possible that older heterosexual individuals have less exposure to such debates than do sexual minority individuals or that even when these individuals have the same level of exposure, they are not affected by same-sex debates to the same degree as are sexual minority individuals. Previous research on this topic has used survey methods, which cannot control for the degree of exposure to such debates. Sexual minority individuals may pay closer attention to debates concerning same-sex marriage than do heterosexual individuals. Future studies should examine whether younger and older individuals are affected by debates concerning same-sex marriage to the same degree and whether heterosexual and sexual minority individuals are exposed to debates concerning same-sex marriage to similar degrees.

Although the current studies have certain limitations, the findings of both of these studies are largely similar and complement each other. First, the legalization of same-sex marriage may involve several sources that may affect people's subjective wellbeing, including public debates concerning same-sex marriage, the symbolic meanings of the legalization of same-sex marriage, and the rights offered to same-sex couples as a result of the legalization of same-sex marriage. It is possible that the current research identified largely similar findings among heterosexual and sexual minority participants (for whom the main effect of condition was significant in Studies 1 and 2) because of its focus on the effects of public debates concerning same-sex marriage. The symbolic meanings of the legalization of same-sex marriage and the rights offered to same-sex couples may affect sexual minority individuals more than heterosexual individuals. These effects may also take specific forms only for sexual minority individuals. Researchers could explore other potential consequences of these factors (e.g., self-acceptance or feelings of injustice) to test these possibilities. Second, participants who exhibited similar demographic characteristics were recruited for the experimental studies conducted for this research, thus precluding the use of a representative sample for Taiwan. Despite the lack of external validity, the findings of this research suggest that young people may indeed suffer or benefit from the arguments that have been made as part of public debates. Third, a paired sample was used in the first study, which is not a common approach in experimental studies. The sample size of the paired sample was relatively small, which may raise questions pertaining to statistical power in this context. However, this paired sample allowed participants who exhibited similar demographic characteristics to be recruited, thus reducing error variance. In addition, the second study, which focused on a large convenience sample, produced similar findings. These two studies thus complement each other and improve the robustness of the conclusions drawn in this research.

Do the findings of this research suggest that public debates concerning social policies such as same-sex marriage or immigration should not be allowed? Certainly not. However, these findings do indicate that public debates must be held in a responsible manner, namely, on the basis of facts and evidence rather than beliefs or preferences. In the case of same-sex marriage, previous researchers have reported various evidence that has indicated large similarities between same-sex and different-sex couples (e.g., see the meta-analyses conducted by Allen and Burrell, 2002; Crowl et al., 2008; Lee, 2019). However, given that unsubstantiated claims (e.g., the claim that vaginal intercourse is the most sanitary form of sex, Liberty Times, 2018) and negative campaigns (Hsieh, 2018) characterized public debates during the time of the aforementioned referenda in Taiwan; the government has a duty to protect its citizens from “harm and injustice” (Riggle et al., 2005).

As society progresses to the point that human rights and equality become norms and are readily accepted by young people, the setbacks resulting from social changes that challenge traditional systems and customs are likely to result in alienation and frustration among young people. The current research highlights such impacts at a time when debates concerning same-sex marriage were taking place in Taiwan. Understanding the impacts of such debates concerning same-sex marriage may help us learn from these lessons and establish a society that respects and accommodates different parties.

## Data Availability

The original contributions presented in the study are included in the article/Supplementary material, further inquiries can be directed to the corresponding author/s.

## References

[B1] Adamczyk A. Cheng Y. H. A. (2015). Explaining attitudes about homosexuality in Confucian and non-Confucian nations: is there a ‘cultural' influence? Soc. Sci. Res. 51, 276–289. 10.1016/j.ssresearch.2014.10.002 25769867

[B2] Allen M. Burrell N. (2002). “Sexual orientation of the parent: the impact on the child,” in Interpersonal Communication Research: Advances Through Meta-analysis, eds. M. Allen and R. W. Preiss (Mahwah, NJ: USum Associates Publishers), 125–143.

[B3] Bai T. (2021). Confucianism and same-sex marriage. Polit. Relig. 14, 132–158. 10.1017/S1755048320000139

[B4] Balsam K. F. Beauchaine T. P. Rothblum E. D. (2008). Three-year follow-up of same-sex couples who had civil unions in Vermont, same-sex couples not in civil unions, and heterosexual married couples. Dev. Psychol. 44, 102–116. 10.1037/0012-1649.44.1.102 18194009

[B5] Benson J. E. Elder G. H. (2011).Young adult identities and their pathways: a developmental and life course model. Dev. Psychol. 47, 1646–1657. 10.1037/a0023833 21668096 PMC3792649

[B6] Berggren N. Bjornskov C. NIlsson T. (2018). Do equal rights for a minority affect general life satisfaction. J. Happiness Stud. 19, 1465–1483. 10.1007/s10902-017-9886-6

[B7] Boertien D. Vignoli D. (2019). Legalizing same-sex marriage matters for the subjective well-being of individuals in same-sex unions. Demography 56, 2109–2121. 10.1007/s13524-019-00822-1 31696409

[B8] Casey L. Wootton B. M. McAloon J. (2020). Mental health, minority stress, and the Australian marriage law postal survey: longitudinal study. Am. J. Orthopsychiatry 90, 546–556. 10.1037/ort0000455 32338941

[B9] Chang Y.-H. Tu S.-H. Liao P.-S. (2014). 2012 Taiwan Social Change Survey (Round 6, Year 1): Gender (C00223_2) [Data file]. Survey Research Data Archive, Academia Sinica. Available at: https://srda.sinica.edu.tw/

[B10] Chen M. H. Ko N. Y. Huang Y. T. Hu H. F. Lu W. H. Yen C. F.. (2021). Poor mental health among Taiwanese people experiencing the public debates on and referendums for same-sex marriage: a Facebook online survey. J. Formos. Med. Assoc. 120, 1069–1079. 10.1016/j.jfma.2020.10.027 33189505

[B11] Chen S. Van Ours J. C. (2022). Mental health effects of same-sex marriage legalization. Health Econ. 31, 42–56. 10.1002/hec.4441 34628683 PMC9293432

[B12] Cheng Y. A. Wu F.-C. F. Adamczyk A. (2016). Changing attitudes toward homosexuality in Taiwan, 1995-2012. Chin. Sociol. Rev. 48, 317–345. 10.1080/21620555.2016.1199257

[B13] Chien C. L. Li M. C. Huang L. L. (2009). Multiple ways to subjective well-being: the divergence and convergence of double self-construals in Taiwan. Chin. J. Psychol. 51, 453–470.

[B14] Chin T.-F. (2020). Addressing heteronormativity: gendered familial appellations as an issue in the same-sex marriage debate in Taiwan. Sexualities 23, 1080–1096. 10.1177/1363460719884022

[B15] Chunghua 21st Century Think Tank (2013). 2012-2013 Taiwan family and marriage survey report.

[B16] Crowl A. Ahn S. Baker J. (2008). A meta-analysis of developmental outcomes for children of same-sex and heterosexual parents. J. GLBT Fam. Stud. 4, 385–407. 10.1080/15504280802177615

[B17] Diener E. Smith H. Fujita F. (1995). The personality structure of affect. J. Pers. Soc. Psychol. 69, 130–141. 10.1037/0022-3514.69.1.130

[B18] Diener E. D. Emmons R. A. Larsen R. J. Griffin S. (1985). The satisfaction with life scale. J. Pers. Assess. 49, 71–75. 10.1207/s15327752jpa4901_13 16367493

[B19] Dion M. L. Diez J. (2017). Democratic values, religiosity, and support for same-sex marriage in Latin America. Lat. Am. Polit. Soc. 59, 75–98. 10.1111/laps.12034

[B20] Douglass R. P. Conlin S. E. Duffy R. D. Allan B. A. (2017). Examining moderators of discrimination and subjective well-being among LGB Individuals. J. Couns. Psychol. 64, 1–11. 10.1037/cou0000187 27929299

[B21] Drabble L. A. Wootton A. R. Veldhuis C. B. Riggle E. D. B. Rostosky S. S. Lannutti P. J.. (2021). Perceived psychosocial impacts of legalized same-sex marriage: a scoping review of sexual minority adults' experiences. PLoS ONE 16:e0249125. 10.1371/journal.pone.0249125 33956825 PMC8101749

[B22] Ecker S. Lykins A. (2023). “Voted yes—What else can I do?”: coping with stigma-related stress during the Australian marriage equality debate. Psychol. Sex. Orientat. Gend. Divers. 10, 324–336. 10.1037/sgd0000543

[B23] Ecker S. Riggle E. D. Rostosky S. S. Byrnes J. M. (2019). Impact of the Australian marriage equality postal survey and debate on psychological distress among lesbian, gay, bisexual, transgender, intersex and queer/questioning people and allies. Aust. J. Psychol. 71, 285–295. 10.1111/ajpy.12245

[B24] Flores A. R. Hatzenbuehler M. L. Gates G. J. (2018). Identifying psychological responses of stigmatized groups to referendums. Proc. Nat. Acad. Sci. 115, 3816–3821. 10.1073/pnas.1712897115 29581304 PMC5899433

[B25] Frost D. M. Fingerhut A. W. (2016). Daily exposure to negative campaign messages decreases same-sex couples' psychological and relational well-being. Group Process. Intergr. Relat. 19, 477–492. 10.1177/1368430216642028

[B26] Frost D. M. Meyer I. H. (2023). Minority stress theory: application, critique, and continued relevance. Curr. Opin. Psychol. 51:101579. 10.1016/j.copsyc.2023.101579 37270877 PMC10712335

[B27] Garrison S. M. Doane M. J. Elliott M. (2018). Gay and lesbian experiences of discrimination, health, and well-being: surrounding the presidential election. Soc. Psychol. Personal. Sci. 9, 131–142. 10.1177/1948550617732391

[B28] Hatzenbuehler M. L. McLaughlin K. A. Keyes K. M. Hasin D. S. (2010). The impact of institutional discrimination on psychiatric disorders in lesbian, gay, and bisexual populations: a prospective study. Am. J. Public Health 100, 452–459. 10.2105/AJPH.2009.168815 20075314 PMC2820062

[B29] Hatzenbuehler M. L. O'Cleirigh C. Grasso C. Mayer K. Safren S. Bradford J.. (2012). Effect of same-sex marriage laws on health care use and expenditures in sexual minority men: a quasi-natural experiment. Am. J. Public Health 102, 285–291. 10.2105/AJPH.2011.300382 22390442 PMC3484969

[B30] Hsieh M. Y. (2018). The darkest day in Taiwan's human rights history! Anti-gay forces won a complete victory in all 5 referenda on same-sex marriage and LGBTQ education. Available at: https://www.storm.mg/article/650119?page=1 (accessed April 25, 2019).

[B31] Huang C. Chang C. C. (2018). Political Polarization Survey (TIGCR-PPS 2018).

[B32] Kavanaugh S. A. Taylor A. B. Stuhlsatz G. Neppl L. Lohman T. K. B. J. (2020). Family and community support among sexual minorities of color: the role of sexual minority identity prominence and outness on psychological well-being. J. LGBT Fam. Stud. 16, 1–17. 10.1080/1550428X.2019.1593279

[B33] Kenny D. A. (1995). The effect of nonindependence on significance testing in dyadic research. Pers. Relatsh. 2, 67–75. 10.1111/j.1475-6811.1995.tb00078.x

[B34] Kuan H.-W. (2019). Marriage equality and legal mobilization: the litigation and legislative actions before Judicial Yuan Interpretation No. 748. Taiwan Found. Democr. 16, 1–44.

[B35] Lee I.-C. (2019). 彩虹家庭的現身:家長與子女表現的整合分析 [The coming-out of rainbow families: a meta-analysis of outcomes for parents and offspring]. J. Womens Gend. Stud. 45, 7–58.

[B36] Lee I.-C. (2020). 性別平權與婚姻平權:從研究結果反思社會正義 [Gender equality and marriage equality: from research findings to reflections upon social justice]. Res. Appl. Psychol. 72, 47–89.

[B37] Lee I.-C. Jou Y.-H. Wong K. Weng S.-Y. Ma L.-H. (in press). From sexual romantic experiences to identity: Understanding the self in the framework of heteronormality. Chin. J. Psychol.

[B38] Lee I.-C. Lin W.-F. (2022). Us versus them: the debates on the legislation of same-sex marriage (1994-2015) in Taiwan. J. Homosex. 69, 655–676. 10.1080/00918369.2020.1848148 33269991

[B39] Lee T.-T. Hicks G. R. (2011). An analysis of factors affecting attitudes toward same-sex marriage: do the media matter? J. Homosex. 58, 1391–1408. 10.1080/00918369.2011.614906 22029563

[B40] Liberty Times (2018). Anti-gay professor's “vaginal sterility theory” has led to heated discussions among gynecologists: a rebuttal. Liberty Times 2018/11/06. Available at: https://news.ltn.com.tw/news/life/breakingnews/2603202 (accessed April 25, 2019).

[B41] Lin H.-C. Chen Y.-L. Ko N.-Y. Chang Y.-P. Lu W.-H. Yen C.-F. (2020). Impacts of public debates on legalizing the same-sex relationships on people's daily lives and their related factors in Taiwan. Int. J. Environ. Res. Public Health 17:8606. 10.3390/ijerph17228606 33228166 PMC7699598

[B42] Lin I.-H. Ko N.-Y. Huang Y.-T. Chen M.-H. Lu W.-H. Yen C.-F.. (2019). Effect of same-sex marriage referendums on the suicidal ideation rate among nonheterosexual people in Taiwan. Int. J. Environ. Res. Public Health 16:3456. 10.3390/ijerph16183456 31533353 PMC6765861

[B43] Lin P.-T. (2020). Who supports same-sex marriage? Exploring the mass bases of the same-sex marriage issue in Taiwan. J. Soc. Sci. Philos. 32, 207–238. 10.53106/1018189X2020063202002

[B44] Mallett R. K. Huntsinger J. R. Sinclair S. Swim J. K. (2008). Seeing through their eyes: when majority group members take collective action on behalf of an outgroup. Group Process. Intergr. Relat. 11, 451–470. 10.1177/1368430208095400

[B45] Meyer I. H. (2003). Prejudice, social stress, and mental health in lesbian, gay, and bisexual populations: conceptual issues and research evidence. Psychol. Bull. 129, 674 −697. 10.1037/0033-2909.129.5.674 12956539 PMC2072932

[B46] Paolini S. McIntyre K. (2019). Bad is stronger than good for stigmatized, but not admired outgroups: meta-analytical tests of intergroup valence asymmetry in individual-to-group generalization experiments. Pers. Soc. Psychol Bull. 23, 3–47. 10.1177/1088868317753504 29473444

[B47] Pinel E. C. Bronson C. A. Zapata J. P. Bosson J. K. (2019). I-sharing after a gender status threat and its implications for attitudes toward gay men. Psychol. Men Masc. 20, 299–309. 10.1037/men0000161

[B48] Prewitt-Freilino J. Bosson J. (2008). Defending the self against identity misclassification. Self Identity 7, 168–183. 10.1080/17405620701330706

[B49] Riggle E. D. Thomas J. D. Rostosky S. S. (2005). The marriage debate and minority stress. PS: Polit. Sci. Polit. 38, 221–224. 10.1017/S1049096505056337

[B50] Rostosky S. S. Riggle E. D. B. Horne S. G. Miller A. D. (2009). Marriage amendments and psychological distress in lesbian, gay, and bisexual (LGB) adults. J. Couns. Psychol. 56, 56–66. 10.1037/a0013609

[B51] Ryan W. Hunger J. M. Major B. (2017). Applying intergroup relations research to understanding LGB health disparities. J. Soc. Issues 73, 477–472. 10.1111/josi.12227

[B52] Stulhofer A. Rimac I. (2009). Determinants of homonegativity in Europe. J. Sex Res. 46, 24–32. 10.1080/00224490802398373 19012059

[B53] Whyke T. W. (2022). Discourses of heteronormativity and power: the ethical position of Confucianism on same-sex behaviour in China. J. Homosex. 70, 1787–1806. 10.1080/00918369.2022.2042663 35213270

[B54] Wong R.-X. (2018). 尤美女:公投後據通報已有9位同志自殺身亡、23件霸凌。 [Yu, Mei-Nu: 9 homosexuals alleged to commit suicide and 23 bullying cases after the referenda.] *ETtoday* 2018/11/30. Available at: https://www.ettoday.net/news/20181130/1319653.htm (accessed April 25, 2019).

[B55] Wu C. H. Yao G. (2006). Analysis of factorial invariance across gender in the Taiwan version of the Satisfaction with Life Scale. Pers. Individ. Dif. 40, 1259–1268. 10.1016/j.paid.2005.11.01235671295

[B56] Yeh T. D.-L. (2017). 良言傷人,六月亦寒:台灣反對同性婚姻網路言論探析 [Good words hurt too: the cyberdiscourse against same-sex marriage in Taiwan]. J. Archaeol. Anthropol. 86, 69–110.

[B57] Yen C.-F. Ko N.-Y. Huang Y.-T. Chen M.-H. Lin I.-H. Lu W.-H.. (2020). Preference about laws for the legal recognition of same-sex relationships in Taiwanese people before and after same-sex marriage referenda: a Facebook survey study. Int. J. Environ. Res. Public Health 17:2000. 10.3390/ijerph17062000 32197386 PMC7142610

[B58] Zhang X. (2018). How should Confucianism view the legalization of same-sex marriage? Int. J. Chin. Comp. Philos. Med. 16, 53–72. 10.24112/ijccpm.161649

