# Supplementary material for: How it affects me: the effects of arguments in public debates on marriage equality for young people in Taiwan
Source: Front Psychol. 2025 Jan 22;15:1462431. doi: 10.3389/fpsyg.2024.1462431 (PMC11794249; doi:10.3389/fpsyg.2024.1462431)
Supplement: Supplementary file 1 [file Data_Sheet_1.docx]

Appendix

Essay in the Yes condition

In recent years, whether same-sex couples are allowed to marry has been vigorously debated domestically and internationally. To date, same-sex marriage has been passed in a dozen countries. Proponents argue that homosexuality is not a mental disorder and that sexual orientation cannot be changed by external forces; coupled with the right that people should be allowed to freely choose who to love, same-sex marriage should be legalized to protect the basic human rights of homosexual individuals. Because the nature of marriage comes from love and commitment, when couples give each other love and commitment, the government should encourage and affirm this. Same-sex couples raise children to the same capacity as different-sex couples; the legalization of same-sex marriage protects the rights of children. The government and legal system should encourage same-sex couples who are willing to maintain long-term relationships by legalizing same-sex marriage.

Essay in the No condition

In recent years, whether same-sex couples are allowed to marry has been vigorously debated domestically and internationally. To date, same-sex sexual acts have been classified as criminal offenses in more than 70 countries. Opponents argue that same-sex couples do not have the ability to naturally have children, so the nature of their relationships is fundamentally different from that of different-sex couples. As a result, the government should not offer them marriage rights. In addition, the legalization of same-sex marriage would challenge the current marital system, violating Chinese social norms, further reducing birth rates, and depriving children of the right to have a father and a mother. The legal system in our country does not forbid same-sex couples from the right to cohabit, so their basic rights are not violated. The government and legal system should not encourage same-sex behaviors, so they should not legalize same-sex marriage.

Essay in the control condition

In recent years, there have been incidents that challenge food safety, such as exposure to plasticizers, the addition of industrial starch to food products, and the use of recycled oil to produce food products, revealing poor food control in our country. Food manufacturers lack conscience, and the government cannot monitor food process procedures. After these incidents, the manufacturers said that they would refund the fees without compensation. The government is called upon to amend the law so that the affected individuals can claim compensation. The government is most criticized for failing to take responsibility for gatekeeping. The officials often acted as if they were the victims and expressed anger and heartache in the media, claiming that they would investigate and severely punish the manufacturers. The government should carefully review the responsibilities they failed to shoulder to curb the recurrence of food safety problems.
